# NG6: Integrated next generation sequencing storage and processing environment

**DOI:** 10.1186/1471-2164-13-462

**Published:** 2012-09-09

**Authors:** Jérôme Mariette, Frédéric Escudié, Nicolas Allias, Gérald Salin, Céline Noirot, Sylvain Thomas, Christophe Klopp

**Affiliations:** 1 Plate-forme bio-informatique Genotoul, INRA, Biométrie et Intelligence Artificielle, BP 52627, 31326, Castanet-Tolosan Cedex, France; 2 Plate-forme genomique Genotoul, INRA, Génétique Cellulaire, BP 52627, 31326, Castanet-Tolosan Cedex, France

## Abstract

**Background:**

Next generation sequencing platforms are now well implanted in sequencing centres and some laboratories. Upcoming smaller scale machines such as the 454 junior from Roche or the MiSeq from Illumina will increase the number of laboratories hosting a sequencer. In such a context, it is important to provide these teams with an easily manageable environment to store and process the produced reads.

**Results:**

We describe a user-friendly information system able to manage large sets of sequencing data. It includes, on one hand, a workflow environment already containing pipelines adapted to different input formats (sff, fasta, fastq and qseq), different sequencers (Roche 454, Illumina HiSeq) and various analyses (quality control, assembly, alignment, diversity studies,…) and, on the other hand, a secured web site giving access to the results. The connected user will be able to download raw and processed data and browse through the analysis result statistics. The provided workflows can easily be modified or extended and new ones can be added. Ergatis is used as a workflow building, running and monitoring system. The analyses can be run locally or in a cluster environment using Sun Grid Engine.

**Conclusions:**

NG6 is a complete information system designed to answer the needs of a sequencing platform. It provides a user-friendly interface to process, store and download high-throughput sequencing data.

## Background

Sequencer manufacturers follow different objectives using different platforms [1]. In the first place they release upgrades of second generation platforms producing more data with updated hardware and sequencing kits. This lowers the sequencing cost per base pair but often focuses these machines on medium or large projects. In the second place, they introduce new laboratory scale platforms such as the Illumina MiSeq or the Roche Junior which target smaller projects. And last, they work on the third generation machines which will not depend on amplified material and therefore get rid of some biases. The first two machines types which are already marketed today associated with a larger scope of sequencing protocols, enabling new studies, push towards more sequencing projects and more users.

Once the sequencing is done, the largest part of the work and the longest time period of the project are dedicated to data analysis. Therefore it is important to provide the new smaller production units and the laboratories in which the projects are conducted with efficient and user-friendly processing environments, enabling quality control and routine analysis. These pieces of software should have several features such as access control, metadata storage on the produced reads, quality control including known bias verification and standard analysis. NG6 was developed to match these goals and to be as flexible as possible, in order to follow sequencing technologies upgrades.

Laboratory information management systems (LIMS) are often focused on the traceability of the biological material. Some of them, such as PIMS [2] or even SLIMS [3], have included extensions to monitor the sequencing process. However few of the open-source LIMS also provide the data processing environment. This feature is present in the galaxy [4] sample tracking module. It is based on the galaxy workflow engine and provides users with an interface to create and track sequencing requests. Once the sequences have been produced, the user can transfer its data files, build and run workflows to process them.

NG6 is an extensible sequencing provider oriented LIMS. It includes read quality control and first level analysis processes which ease the data validation made jointly by the sequencing facility staff and the end-users. It provides a secured user-friendly interface to visualize and download the raw sequences files and the analysis results.

## Implementation

**Figure 1:**
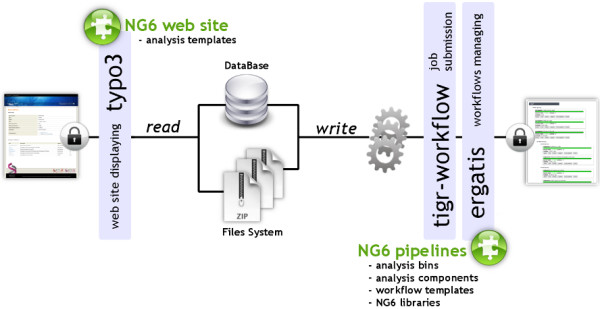
**Architecture of the ng6 application.** NG6 pipelines are available within the ergatis workflow environment. The analyses are processed either on a local system or on a distributed environment. While the analyses are running, they store the resulting files on the file system and add information about the run in the database. The produced data is then displayed by the NG6 extension of the tyo3 CMS. Both NG6 web site and NG6 pipelines are accessible through a web browser after authentication.

NG6 can be split into two distinct parts: the pipelines and the web site (Figure 1). The pipelines gather a set of analyses adapted to the produced sequences. They can only be accessed and launched by the sequencing facility team. The pipelines are running in Ergatis [5]: a workflow management system able to iterate through multiple inputs in order to run them at the same time on a computer farm. These jobs perform analysis and save the analysis results in the NG6 database and directories. The web site part, presenting the results has been implemented as a typo3 [6] extension.

NG6 uses three data types: project, run and analysis. A project is a collection of runs and analysis. A run contains one or several raw files which can be used as inputs of different analysis. A project is owned by a user group and only users within this group are allowed to browse and download data related to this project.

### Building and running pipelines

Pipelines are defined by a set of connected ergatis components. Depending on the links between the components, they are processed in a parallel or a serial manner. Most components available in NG6 combine a processing step and a storage step. This last one stores, on one hand, resulting files into the ad-hoc directory structure and, on the other hand, saves information into the database such as software version, parameters, links between analysis and resulting figures.

In the current version, NG6 offers a set of pipelines adapted to two platforms (Roche 454, Illumina HiSeq), four file formats (sff, fastq, fasta and qseq) and handles both casava 1.7 and casava 1.8 outputs of the illumina package [7]. It includes analyses such as quality control, genomic read alignment, BAC assembly, 16S/18S diversity analysis, expression quantification using 16S amplicons. In order to handle multiplexed runs, some pipelines first split the input read file into sample files, process and collect results on each of them and last merge these results in a summary table.

As an example, the 454_default pipeline processes sff files, coming from the Roche sequencer. It first performs usual statistical analysis on the reads, then tracks down contamination from common contaminant databases (ecoli, yeast and phage) using blast [8] returning a list of contaminated sequence IDs. Contamination between the different regions is also traced using the sfffile script included in the Roche Newbler package [9]. Sequences with incorrect MID (Multiplexed ID) are discarded and the number of contaminated sequences is returned to the end-user. Roche 454 sequencing kits include control fragments known as spike-ins within each run. Statistics on the corresponding sequences are used to check if the run matches the expected quality standard. In the next step reads are cleaned using the pyrocleaner script [10]. It discards reads considering different criteria such as length, base quality, complexity, number of undetermined bases, multiple copy reads or even faulty paired-ends. The analysis results are presented to the users in a summary table. Last, a de novo assembly is performed on the cleaned reads using the Newbler runAssembly command [9]. Some basic figures regarding the assembly results, such as contig count, N50 value, contig length distribution or even contig length versus sum of read length per contig diagram are presented to the user in order to ease the assembly quality assessment.

When the pipeline execution is over, all analysis and runs newly added to the system are flagged as hidden. This was meant to permit the validation of the run by the team in charge of the sequencer before data release to the end-user.

**Figure 2:**
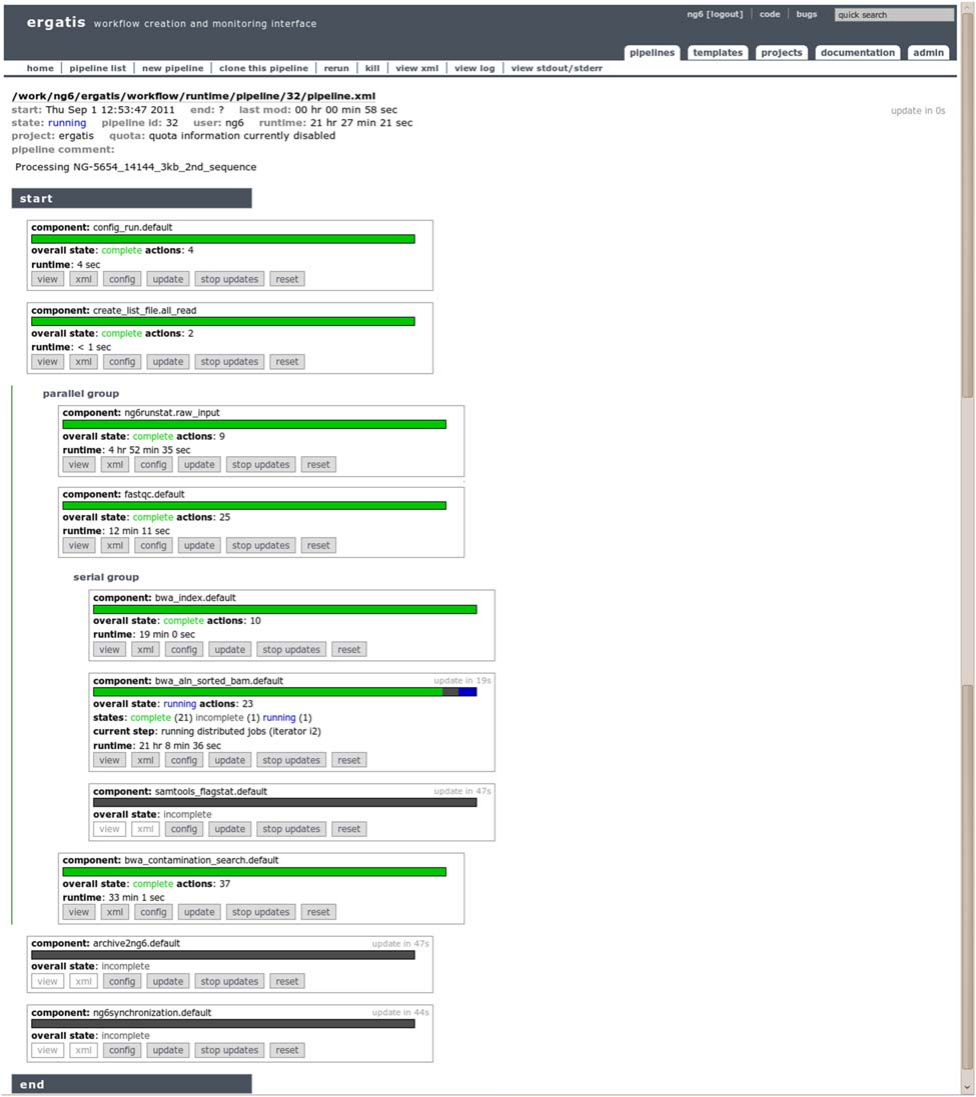
**Executing and monitoring workflows.** To monitor, execute and create pipelines, NG6 relies on the ergatis workflow management system. This figure presents a pipeline running on illumina data and producing an alignment against a reference genome, some statistics on the reads, some statistics on the alignment produced and on the sought contamination.

NG6 also provides two components enabling to start a pipeline with data already loaded into the system. The ng6run2ergatis component takes a run ID and a file pattern in order to create an input file list which can be used as input for other components. The same can be done with the ng6analysis2ergatis component to work on previous analysis result files. This enables to launch new pipelines on datasets already stored in the system in order to answer new requests. When building a new pipeline, the administrator will have the choice between several already available components such as cleaning tools : smartkitcleaner, adaptatorcleaner, 16Scleaner or cutadapt [11], alignment tools : bwa [12,13], blast, statistical tools : fastqc [14], the samtools [15], 16S/18S diversity assessment tools as mothur [16] or other utilities as fastq_extract or sff_extract [17]. After the configuration step, the administrator will be able to run the pipeline and monitor the processing steps states (Figure 2).

The analyses provided in NG6 have been designed to limit the used disk space and the number of temporary files. As an example, the bwa alignment against a reference genome, performed on illumina reads, chains bwa and samtools using the unix pipe command.

A cluster environment has often a local optimized file system. NG6 moves files from the cluster file system to the storage file system using the ng6synchronization component. Until synchronization is completed, a warning message is displayed to inform the end-user.

### Browsing and downloading results

**Figure 3:**
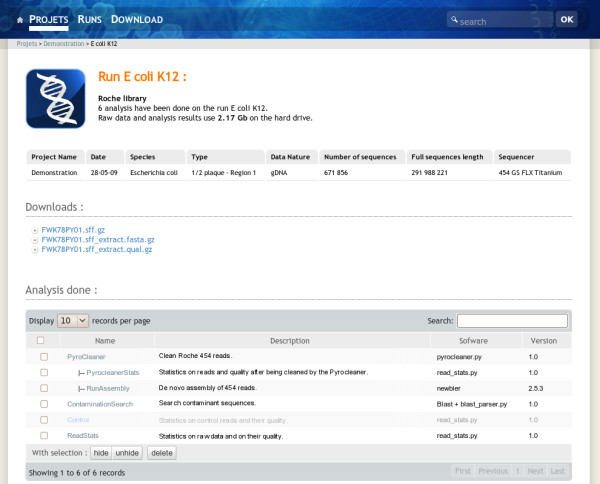
**Administrator view of a run.** The administrator view enables multiple analysis selection in order to hide, unhide or delete the selected elements. Once hidden, an analysis will no longer be displayed to the end-user. As an example, the Control analysis is displayed as hidden, so this one will not be displayed in the end-user view.

A user can access his projects or runs using the menu bar items at the top of the page. The project and run links list all projects and runs he has access to. Once in a project, the user will see all the related runs and analysis performed on the project level. At the run level the system displays corresponding metadata such as species, sequence type and data volume. It also gives access to the sequence files and hierarchically lists analysis performed on the run. The analysis view displays analysis results and provides a direct access to the resulting data files (Figure 3). At each level, the NG6 interface shows the used disk space. The download manager accessible from the menu bar permits to select and download data and analysis results files. To avoid data duplication, if the user has an unix account on the NG6 server, the software provides the possibility to create symbolic links between the data files and his home directory.

As a typo3 plug-in, NG6 can easily be included in any web site built with this CMS. The NG6 plug-in is compliant with the national language support system of typo3. Configuring the system for a new language only consists in translating and adding the corresponding language files. So far, only English and French are supported.

### Right accesses and administration

NG6 offers two user status : administrator and end-user and two data access levels : public and private. Within each level the items can be hidden or unhidden. This allows to manage access rights considering the user type (Table 1). NG6 uses the typo3 user tables and management system. Rights are given on a project level to a user group. A user can be part of multiple groups. Once the user is logged on the web site, he can only browse projects of his groups.

**Table 1:** Users and data right management

	**Data right level**			
	**Public**		**Private**	
	**Hidden**	**Unhidden**	**Hidden**	**Unhidden**
Project administrator	✓	✓	✓	✓
Connected user	*✗*	✓	✗	✓
Unconnected user	*✗*	✓	✗	✗

Considering a specified project, the administrator can browse all the runs and analysis linked to it. He is the only one with write accesses and the only one able to hide, unhide or publish a project. A connected user can browse all projects, runs and analysis that have been unhidden by the administrator. An unconnected user has only access to public projects if those ones are unhidden

**Figure 4:**
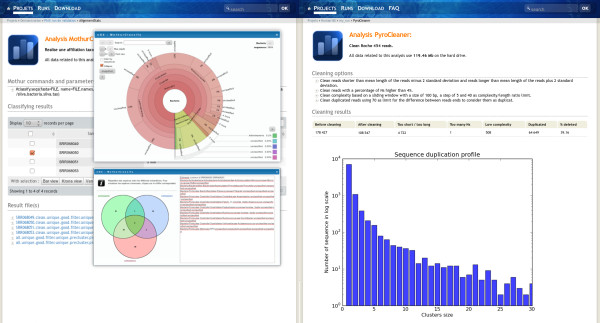
**Example of some analysis view**. The analysis result layout is defined in a smarty template, thus enabling different layouts for the end-user. Figure five shows examples of MothurClassify and PyroCleaner analysis result displays.

The project administrator has all rights on the project, he can delete, hide, unhide, publish and unpublish the whole project with related runs and analysis. A hidden project is only visible to the project administrator, this was designed in order to permit the validation of the run by the team in charge of the sequencer before releasing the data to the end-user. To give access to the project, once the data is validated, the administrator unhides it. This is also true for analysis (Figure 4). The metadata fields are editable on line by the administrator.

A published project is openly accessible on the web site. For example, you can access our demonstration project using the following link : http://ng6.toulouse.inra.fr/index.php?id=3. This feature provides the biologists with a fast and easy way to make their data accessible to their community.

### Adding new analysis

NG6 web site is a Typo3 extension written in php. It uses the smarty template engine [18] and the jquery javascript library [19]. Adding new analysis into NG6 requires three steps. The first one is writing the ergatis component of the analysis. Each parameter, input and output required by the analysis has to be specified in the configuration file. Second, a simple python script has to be programmed using the NG6 API and the provided skeleton to define the data stored in the database and the result files stored in the directory structure. Finally, a smarty template is specified to set the corresponding analysis display. While writing the smarty template, the developer has access to several objects to build the analysis display as wished. Several HTML classes are available to ease javascript functionalities implementation.

## Results and discussion

NG6 has been in production since September 2009 at the genomic platform of GenoToul [20] and stores more than 950 runs corresponding to 96 projects and using 5 TB on the hard drive. The system stores Illumina and Roche 454 runs produced by different sequencer versions. Pipelines are configured and launched by the genomic platform staff for one year.

Assessing the quality of the produced reads is an important task for a sequencing center. Making it automatic saves a lot of time. Displaying the analysis results within a user-friendly interface eases the discussions with the end-users.

Other read analysis environments are available to biologists. The most popular today is Galaxy. We have chosen to implement our own system because Galaxy and NG6 target different aims and focus on different users. Galaxy aims at simplifying data processing for researchers. It includes modules processing sequencing data. NG6 is a sequencing provider focused LIMS gathering specialized pipelines and website.

## Conclusions

NG6 is an information system providing a set of automated analysis pipelines built to process NGS (Next Generation Sequencing) data which can be executed locally or in a cluster environment. It is built upon well documented and extensively used components such as ergatis and typo3. The current version of NG6 offers several pipelines but some others are under-construction: RNAseq using tophat [21] and cufflinks [22] and miRNA expression analysis.

## Availability and requirements

The NG6 code is freely available on the web. To ease the installation, the package and all its dependencies are also available as a virtual machine. Installing and maintaining the system would require expertise in Linux system administration. The project is hosted in a forge environment in order to open it to the developers community.

· Project name: ng6

· Project home page: https://mulcyber.toulouse.inra.fr/plugins/mediawiki/wiki/ng6/index.php/Main_Page

· Operating system(s): Platform independent

· Programming language: Python/PHP

· Other requirements: VMWare or VirualBox

· License: GNU GPL

· Any restrictions to use by non-academics**:** none

## Competing interests

The authors declare that they have no competing interest.

## Authors’ contributions

JM and CK conceived and designed the project. JM, FE, GS, CN, NA implemented NG6 pipelines and web site. JM and ST packaged NG6 into a virtual machine. JM and CK drafted the manuscript. All authors read and approved the final manuscript.

## References

[B1] Glenn TC Field guide to next-generation DNA sequencers Mol Ecol Resour 2011 11 759 769 10.1111/j.1755-0998.2011.03024 10.1111/j.1755-0998.2011.03024.x 21592312

[B2] Troshin PV Vincent LG P Denise A Baldwin SA McPherson MJ Barton GJ PIMS sequencing extension: a laboratory information management system for DNA sequencing facilities BMC Research Notes 2011 4 48 10.1186/1756-0500-4-48 10.1186/1756-0500-4-48 21385349 PMC3058032

[B3] Van Rossum T Tripp B Daley D SLIMS—a user-friendly sample operations and inventory management system for genotyping labs Bioinformatics 2010 26 14 1808 1810 10.1093/bioinformatics/btq271 20513665 PMC2894515

[B4] Giarine B Riemer C Hardison RC Burhans R Elnitski L Shah P Shang Y Blankenberg D Albert I Taylor J Miller W Kent WJ Nekrutenko A Galaxy: A platform for interactive large-scale genome analysis Genome Res 2005 15 1451 1455 10.1101/gr.4086505 16169926 PMC1240089

[B5] Orvis J Ergatis: a web interface and scalable software system for bioinformatics workflows Bioinformatics 2010 10.1093/bioinformatics/btq167 10.1093/bioinformatics/btq167 PMC2881353 20413634

[B6] Typo3 web sitehttp://typo3.org/

[B7] Illumina web site http://www.illumina.com/

[B8] Altschul SF Gish W Miller W Myers EW Lipman DJ Basic local alignment search tool J Mol Biol 1990 215 3 403 410 2231712 10.1016/S0022-2836(05)80360-2

[B9] Roche 454 web site http://www.my454.com/

[B10] Mariette J Noirot C Klopp C Assessment of replicate bias in 454 pyrosequencing and a multi-purpose read-filtering tool BMC Research Notes 2011 4 149 10.1186/1756-0500-4-149 21615897 PMC3117718

[B11] Cutadapt web site http://code.google.com/p/cutadapt/

[B12] Li H Durbin R Fast and accurate short read alignment with Burrows-Wheeler transform Bioinformatics 2009 25 1754 1760 10.1093/bioinformatics/btp324 19451168 PMC2705234

[B13] Li H Durbin R Fast and accurate long-read alignment with Burrows-Wheeler transform Bioinformatics 2010 26 5 589 595 [PMID: 20080505] 10.1093/bioinformatics/btp698 20080505 PMC2828108

[B14] Fastqc web site http://www.bioinformatics.bbsrc.ac.uk/projects/fastqc/

[B15] Li H Handsaker B Wysoker A Fennell T Ruan J Homer N Marth G Abecasis G Durbin R 1000 Genome Project Data Processing Subgroup: The Sequence alignment/map (SAM) format and SAMtools Bioinformatics 2009 25 2078 2079 [PMID: 19505943] 10.1093/bioinformatics/btp352 19505943 PMC2723002

[B16] Schloss PD Introducing mothur: Open-source, platform-independent, community-supported software for describing and comparing microbial communities Appl Environ Microbiol 2009 75 23 7537 7541 10.1128/AEM.01541-09 19801464 PMC2786419

[B17] Sff_extract web site http://bioinf.comav.upv.es/sff_extract/

[B18] Smarty template engine web site http://www.smarty.net/

[B19] Jquery web site http://jquery.com/

[B20] GenoToul web site http://get.genotoul.fr/

[B21] Trapnell C Pachter L Salzberg SL TopHat: discovering splice junctions with RNA-Seq Bioinformatics 2009 1;25 9 1105 1111 10.1093/bioinformatics/btp120 PMC2672628 19289445

[B22] Roberts A Pimentel H Trapnell C Pachter L Identification of novel transcripts in annotated genomes using RNA-Seq Bioinformatics 2011 10.1093/bioinformatics/btr355 10.1093/bioinformatics/btr355 21697122

